# ‘From Challenges to Confidence’: A Quality Improvement Project by Bradford District Care Foundation Trust IMG Network in Developing Workshops on Leadership, Portfolio Management, Reflective Practice, Coaching and ARCP Preparation for the Career Progression of IMGs

**DOI:** 10.1192/bjo.2026.11427

**Published:** 2026-06-30

**Authors:** Kamran Mahmood, Anilkumar Pillai, Parvathy Ravikumar

**Affiliations:** 1 Bradford District Care Foundation Trust, Bradford, United Kingdom; 2 Leeds and York Partnership Foundation Trust, Leeds, United Kingdom

## Abstract

**Aims:**

The Bradford District Care NHS Foundation Trust IMG Network delivered a face-to-face workshop for International Medical Graduates (IMGs) on 29 September 2024, followed by a virtual workshop via MS Teams on 12 September 2025.

Both sessions focused on supporting IMGs in understanding career progression, one of the eight key pillars of IMG support. The workshop content was informed by the framework outlined in the School of Psychiatry Yorkshire and Humber Deanery’s *Life in the UK as an IMG* *–* *A Psychiatrist’s Journey*, ensuring alignment with regional expectations.

This Quality Improvement project built upon the 2024 in-person workshop to expand its impact. The primary aim was to determine whether a virtual format could improveattendance, offer greater accessibility, reduce costs, and compare favourably with in-person approach. By evaluating engagement and perceived value across both formats, the project explored whether virtual delivery could serve as a sustainable model for ongoing IMG support.

**Methods:**

Focused areas of the workshop remained the same namely leadership, portfolio management, reflective practice, coaching and preparing for ARCP.

Workshop delivery transitioned from in-person to virtual (via Microsoft Teams).

Pre-and post-workshop surveys were conducted to quantify attendee experience and evaluate learning outcomes.

Cost to the trust was compared from 2024 in-person workshop with 2025 online workshop.

**Results:**

Direct costs for the 2025 virtual workshop were minimal and difficult to quantify. However, virtual delivery eliminated venue booking costs, avoided travel and mileage claims and reduced carbon footprint. For comparison, the 2024 in-person workshop cost was £1750 (venue and related expenses).

There was an increased average attendance from 10 (2024) to 26 (2025).

**Conclusion:**

Virtual model of delivering workshop via MS Teams was more cost-effective, environmentally sustainable, and generated better attendance with wider regional participation without compromising on the educational quality of the workshop as demonstrated by post-workshop survey feedback.

Positive post-workshop feedback also supported and favoured continued provision of virtual IMG development workshops in comparison with in-person workshop.

